# Analysis of Driving Behavior Based on Dynamic Changes of Personality States

**DOI:** 10.3390/ijerph17020430

**Published:** 2020-01-08

**Authors:** Fanyu Wang, Junyou Zhang, Shufeng Wang, Sixian Li, Wenlan Hou

**Affiliations:** 1 College of Transportation, Shandong University of Science and Technology, Huangdao District, Qingdao 266590, China; fanyuwang314@163.com (F.W.); junyouzhang@sdust.edu.cn (J.Z.); gsypsbyw666@163.com (S.L.); 2 College of Foreign Language, Shandong University of Science and Technology, Huangdao District, Qingdao 266590, China; wenlanhou314@163.com

**Keywords:** dynamic personality, driving behavior, personality baseline, K-means clustering algorithm, simulated scenarios

## Abstract

This study investigated the relationship between personality states and driving behavior from a dynamic perspective. A personality baseline was introduced to reflect the driver’s trait level and can be used as a basic reference for the dynamic change of personality states. Three kinds of simulated scenarios triggered by pedestrian crossing the street were established using a virtual reality driving simulator. Fifty licensed drivers completed the driving experiments and filled in the Neuroticism Extraversion Openness Five-Factor Inventory (NEO-FFI) questionnaire to measure the drivers’ personality baselines. Key indicators were quantified to characterize the five types of personality states by K-means clustering algorithm. The results indicated that the high-risk situation had a greater impact on the drivers, especially for drivers with openness and extroversion. Furthermore, for the drivers of extroverted personality, the fluctuation of personality states in the high-risk scenario was more pronounced. This paper put forward a novel idea for the analysis of driving behavior, and the research results provide a personalized personality database for the selection of different driving modes.

## 1. Introduction

Motor vehicle traffic accidents and fatalities represent a serious problem in terms of road traffic safety. In China, it was reported that there were 58,091 fatalities and 227,438 people injured in 216,178 motor vehicle traffic accidents recorded in 2018. These accidents led to estimated direct economic costs (e.g., property damage and medical costs) of USD 188 million [1].

Previous studies have revealed that a large percentage of motor vehicle traffic accidents are caused by drivers [2,3,4]. Therefore, research on drivers’ driving behavior has always been a hot spot in the field of road traffic safety [5]. Personality traits have long been recognized as important individual factors that are closely linked with risky driving behaviors and traffic accidents [6,7,8,9]. The theory of personality traits believes that traits are the basic characteristics that determine individual behavior [10,11]. For each driver, personality traits remain relatively stable; however, when affected by a specific driving situation, there will be a certain change of personality state, which is known as dynamic personality [12,13,14,15]. Accordingly, studying the relationship between dynamic personality states and driving behavior in specific driving situations may help to comprehensively understand the reasons for different driving performance of individuals, so as to provide new insight into the design of driving education and accident prevention interventions, and to establish a personalized personality database for the selection of driving modes.

Self-reported questionnaires are often used to analyze the correlation between personality and specific driving performance [16,17,18,19,20,21,22]. Using the scales for measuring personality traits and risky driving behaviors, Zhang et al. found that agreeableness and conscientiousness were negatively correlated with hostile aggression and acts of revenge committed by the drivers themselves, while neuroticism was positively correlated with aggressive driving [23]. Dahlen et al. analyzed 308 drivers and found that drivers with low agreeableness were more likely to engage in aggressive driving behavior [24]. Allowing for the fact that the correlation research to date has completely relied on self-reported results, the authenticity and accuracy of these questionnaire results should be further considered. To solve this problem, kinematic parameters recorded in a real situation joined with a self-reported questionnaire were used to analyze driving behavior [25]. Eboli et al. investigated the correlation between drivers’ conditions and their driving behavior using the questionnaire and the driving data of 30 paths. They found that self-reported results could not be reliable; an objective measure including not only abstract parameters but scales gave more reliable results [26].

As the research in this area has progressed, researchers have further attempted to analyze the relationship between personality and driving behavior in typical situations. However, it is hard to obtain large amounts of data owing to the danger of some scenarios in actual driving environments. Considering that it can simulate actual driving scenarios and eliminate the interference of external factors, virtual reality driving simulators have been used to collect driving data [27,28,29,30]. Using a driving simulator, Linkov et al. designed three driving scenarios with different speed limits to evaluate the relationship between driver personality and their speed. They found that the correlation between speed and conscientiousness was significant and consistent with the results of previous questionnaires [31]. Zicat et al. characterized driving ability using driving speed indicators on a simulator, and the results showed a significant relationship between the anxious, angry personality traits and the speed of young drivers [32]. Riendeau et al. attempted to evaluate the relationship between personality and objective driving results based on simulated driving data. They found that extroversion and neuroticism were significantly correlated with unsafe driving [33]. However, they only considered the connection between speed and personality, and ignored the driver’s own responsiveness and other vehicle indicators except for speed. In this paper, a risk-free simulated driving scenario was first established to quantitatively analyze the correlation between the representative indicators and personality using the cluster analysis model. The driving indicators used included the reaction ability and two indicators derived from speed. The K-means clustering method was chosen as the clustering learning algorithm, as it allows scalability and efficiency to be maintained when dealing with large datasets [34,35].

Mainstream personality research still focuses on the study of non-dynamic and inter-group differences, which is called the traditional approach to personality [36,37]. This approach can be useful in studying the influence of personality on behavior, but neglects the dynamic changes of personality states. In order to solve this problem, psychologists introduced the dynamic system into personality research, including a set of interactive personality elements that lead to the effective and cognitive performance of behavior and characteristics [38,39]. Sosnowska et al. proposed a comprehensive method of personality expression to coordinate the dynamic and stable aspects of personality based on the personality dynamics model. This method captures a trajectory of personality state through three parameters: baseline, variability, and attractiveness. This dynamic approach to personality offers a consensual paradigm of personality with the potential to advance our understanding and knowledge of individual differences [36]. However, the researches only proposed this dynamic theoretical framework, which has yet to be introduced into the field of traffic safety. Therefore, the dynamic personality theory in psychology was grafted to the analysis of driving behavior in this paper. Considering that dynamic personality is closely related to situation and time [40,41], and specific scenarios have different influences on drivers’ personality states, we chose the risk scenarios evoked by a pedestrian crossing the street as the examples for analysis.

The specific objectives of this study were as follows.

- In the risk-free scenario, personality baselines were firstly measured by the NEO-FFI questionnaire. We aimed to establish the correspondence between driving indicators and the “Big Five” personality traits in a quantitative manner using the K-means clustering method.
- In the risk scenarios, the objective was to analyze the influence of specific driving situations and time on the personality states of different drivers from a dynamic perspective, combined with the thresholds of each indicator.

The remainder of the paper is organized as follows. Section 2 introduces the sample, NEO-FFI scale, and K-means clustering theory, and presents the processes used to analyze driving behavior. Section 3 describes the design of the risk-free and high- and low-risk simulated driving scenarios. In Section 4, the thresholds of three indicators corresponding to each personality were first obtained using the K-means clustering method, based on the actual driving data in the risk-free scenario. Secondly, the dynamic approach to personality was integrated into the research method to analyze the changes of each driver’s personality states with situation and time in the high- and low-risk scenarios. Finally, the conclusion is presented in Section 5.

## 2. Methods

### 2.1. Sample

The participants were 50 professional drivers who had a motor vehicle driver’s license. They were recruited through researchers’ personal contacts and through advertisements on the internet. Participants included 29 male and 21 female drivers, and had a mean age of 35.4 years (SD = 10.1). One participant was not included in the analysis because he completed only part of the study due to simulator sickness.

### 2.2. NEO-FFI

At present, the Big Five personality traits are often measured using the NEO Personality Scale; these include neuroticism (N), extroversion (E), openness (O), agreeableness (A), and conscientiousness (C) [42]. The specific characteristics are described in Table 1. Currently, there are two commonly used versions of the scale: NEO-PI-R (Revised Neuroticism Extraversion Openness Personality Inventory) and NEO-FFI (Neuroticism Extraversion Openness Five-Factor Inventory). NEO-PI-R contains 240 items, while NEO-FFI is simplified from NEO-PI-R through the analysis of project factor and has 60 items [43]. Both of them are significantly related, equally reliable and effective, and are widely used as personality rating scales all over the world. Since there are fewer test items and the evaluation time is shorter, we used NEO-FFI to determine the personality baselines of drivers.

There are 60 items on the NEO-FFI scale, 12 for each personality trait, and each item is answered using a five-point Likert scale, ranging from “strongly disagree” to “strongly agree”. Cronbach’s alphas were 0.65 for neuroticism, 0.80 for extraversion, 0.55 for openness, 0.71 for agreeableness, and 0.85 for conscientiousness [44,45]. The purpose of this questionnaire was to measure the driver’s personality baseline, which reflects the person’s trait level and can be used as a basic reference point for evaluating the dynamic change of personality states [36].

### 2.3. The Theory of K-Means Clustering

Based on the principle of distance similarity, the K-means clustering algorithm divides two groups of samples with small distance into the same cluster, and finally forms different clusters from all the sample data with similar distances to obtain compact and independent categories [34]. First, k groups of initial clustering centers are randomly selected from the input data set, and then, according to the principle of distance proximity, the appropriate distance formula is used to calculate the distance between each data object and the k cluster centers, after which the data are divided into the cluster domains where the nearest cluster centers are located. Finally, a cluster composed of the cluster center and all data objects assigned to the center is formed. After all data are allocated successfully once, the average value of all data objects in each cluster is calculated repeatedly to obtain the new cluster center, i.e., k group clustering is obtained.

The termination conditions of the algorithm are as follows.

1. There is no or minimum number of data objects reassigned to different clusters;
2. There is no or minimum number of clustering centers changing again;
3. The square of the local error is the smallest.

The calculation formula of error sum of squares J is shown in Equation (1). The effect of clustering is negatively correlated with the value of J. The smaller the value is, the better the clustering effect of the data is.

(1) $$J=\sum_{j=I}^{k}\sum_{u\in c_{j}}d\left(X_{u},m_{j}\right)$$

$m_{j}$, representing the clustering center vector, is represented by Equation (2).

(2) $$m_{j}=\frac{1}{N_{j}}\sum_{u\in c_{j}}X_{u}$$

where $X_{u}$ is the vector of all attributing values of data u; $m_{1},m_{2},\cdots m_{k}$ is the vector set corresponding to the clustering center of group k; $c_{j}$ is the clustering domain of clustering center $m_{j}$; and $N_{j}$ is the number of all the data in the clustering domain $c_{j}$.

After comprehensive analysis, a state wherein the cluster centers are maintained unchanged is chosen as the termination condition of the algorithm, where the vector distance is obtained by calculating the distance between the data and the centroid.

There are many distance formulas, such as Euclidean distance and cosine distance. The selection of different calculation formulas will influence the results of distance calculation, thus affecting the clustering effects. The idea of Euclidean distance comes from the actual distance between two points in Euclidean space, which is a common rule of distance measurement. In the case of data with low dimensionality, the Euclidean distance algorithm can classify quickly because of its simple algorithm. In this paper, considering that the clustering dimension was only three dimensions, the Euclidean distance was adopted.

(3) $$d(A,B)=\sqrt{{\left(x_{1}-x_{2}\right)}^{2}+{\left(y_{1}-y_{2}\right)}^{2}+{\left(z_{1}-z_{2}\right)}^{2}}$$

where $A=(x_{1},y_{1},z_{1})$, $B=(x_{2},y_{2},z_{2})$, and d(A,B) is the distance between the two points A and B in Euclidean space.

### 2.4. Analysis of Driving Behavior

We designed the simulated driving scenes according to the phenomenon of a pedestrian crossing the street in a real driving environment, and invited the drivers to carry out the simulated driving experiments and to fill in the NEO-FFI scale. Dozens of parameters were recorded in the virtual reality driving simulator, including time, driving speed, the value of steering velocity, etc. After the comprehensive analysis of all the driving data, the indexes with large significant differences were selected. The thresholds of driving indexes corresponding to the Big Five personality states were then obtained using the K-mean clustering model. Based on the thresholds, we analyzed the changes of personality states in different risk scenarios.

**Figure 1:**
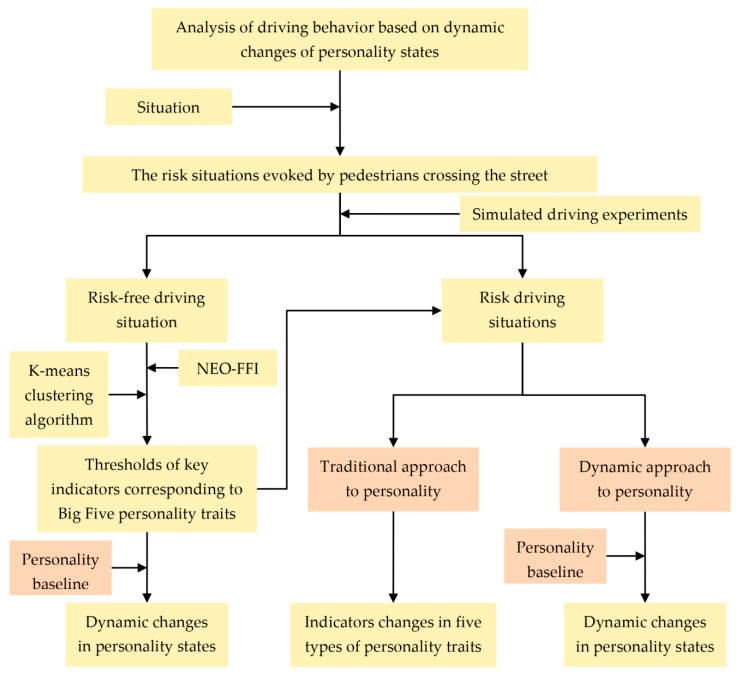
The processes used to analyze the correlation between personality states and driving behavior. Note: NEO-FFI: Neuroticism Extraversion Openness Five-Factor Inventory.

We designed a set of complete processes to analyze the correlation between personality states and driving behavior from the dynamic perspective, as is shown in Figure 1.

## 3. Design of the Experiments

In this paper, the simulated experiments were carried out using the UC-win/Road.13.0 driving simulator at the Intelligent Transportation research center, Shandong University of Science and Technology. The driving simulation hardware equipment consisted of three networked computers and interfaces such as steering system, pedal, automatic shift, etc. The traffic environment was projected onto a large visual screen, which consisted of three sub-screens providing a driving view of 135 degrees. The resolution of the scene was 1920 × 1080, and the refresh rate ranged from 20 to 60 Hz depending on the complexity of the traffic environment. The simulator recorded changes of horizontal and longitudinal driving indexes such as position coordinates, speed, and acceleration of the target vehicle. According to the theory of dynamic personality, we used the dynamic changes of indexes to reflect personality states.

### 3.1. Design of Typical Driving Scenarios

The experimental scenarios included risk-free and risk scenarios, in which the road was a two-lane urban road with high visibility and low traffic flow. According to the degree of conflict between vehicle and pedestrian, the risk scenarios were divided into low- and high-risk scenarios. The design of the three kinds of scenarios was as follows.

#### 3.1.1. Risk-Free Scenario

**Figure 2:**
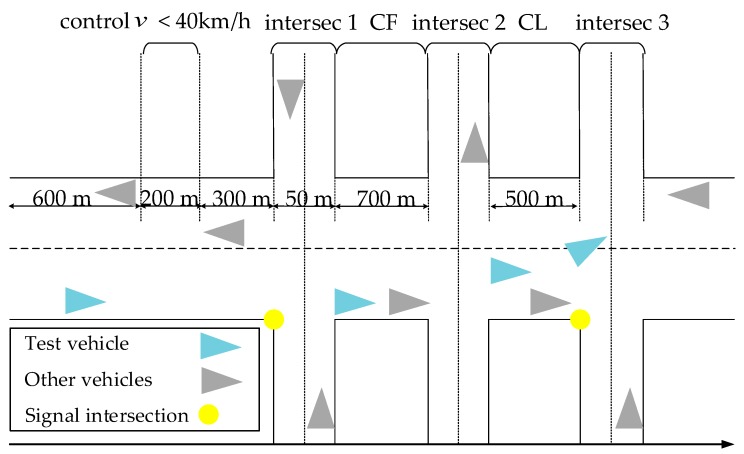
Schematic diagram of risk-free scenario.

The experiment in the risk-free scenario was used to record the basic vehicle control ability and the reaction ability of drivers in a normal driving environment. The road was a four-lane dual carriageway with a lane width of 3.5 m, and the free traffic flow of the road was set to simulate the actual road conditions. Specifically, the maximum speed in the speed limit segment was 40 km/h, while it was 60 km/h for the other segments. The segment settings in the risk-free scenario are shown in Figure 2, and “intersec1”, “2”, and “3” represent the first, second, and third intersection, respectively. “CF” stands for car following, “CL” for lane changing, and the instructions for CL and CF appeared randomly.

#### 3.1.2. Risk Scenarios

In the high-risk scenario, the blocking of the right obstacle (the bus parked in front of the station) caused a blind area for drivers in the process of driving. When the pedestrian crossed the road in front of the bus, the driver was at high risk due to the lack of warning and their fast speed. In the low-risk situation there was no obstacle obstruction for drivers, so the risk of interaction with the pedestrian was lower than that in the high-risk scenario. The specific scenario settings were as follows.

1.  Low-risk scenario

**Figure 3:**
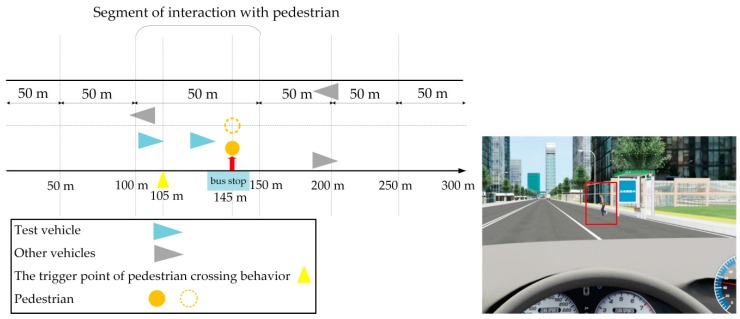
Schematic diagram of low-risk scenario.

The design of the scenario is shown in Figure 3, and the experiment was set as follows: at the beginning of the experiment, the driver started to accelerate with a speed limit of 50 km/h. When the driver reached 105 m, the pedestrian was triggered to cross the street horizontally from the bus stop at a speed of 2 m/s. At this time, the driver was supposed to adjust their driving behavior. After interacting with the pedestrian, the driver continued to drive forward.

2.  High-risk scenario

**Figure 4:**
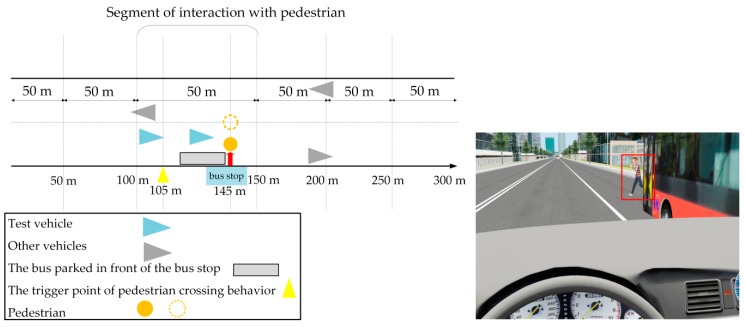
Schematic diagram of high-risk scenario.

The specific design is shown in Figure 4: a bus with a width of 2.5 m was parked in front of the bus stop at 145 m, with the right side of the bus 0.2 m away from the kerbside; that is, the pedestrian walked 2.7 m in front of the bus. The width of the experimental vehicle was 2 m. Similarly to the low-risk scenario, at the beginning of the experiment, the driver also accelerated to 50 km/h, and when the car reached 105 m, the pedestrian crossed the road in a straight line with a uniform speed of 2 m/s from the bus stop. That is to say, it took 1.35 s for the pedestrian to pass the cover of the bus and enter the driver’s field of vision. At this time, the driver was supposed to adjust their specific driving behavior so as to avoid conflict.

### 3.2. Experimental Procedure

Volunteers participated in the driving experiments on the driving simulator. First, the research staff explained the design and purpose of the driving experiments to the drivers. In order to eliminate the unrealistic feeling of the simulated scene, the drivers participated in adaptive training and driving situational immersion training before the formal experiment to help them integrate into the simulated driving environment, and then filled in the NEO-FFI questionnaire. After this warm-up drive, participants completed the risk-free, high-risk, and low-risk simulated driving tests and, at the same time, the staff recorded the drivers’ corresponding driving behaviors in real time. The duration time of a complete experiment was 1 h.

## 4. Discussion

### 4.1. Risk-Free Driving Scenario

After the integrity analyzing and filtering processing of the original driving data collected from the risk-free scenario experiments, 646 groups of available sample data were obtained. SPSS.24 was used to analyze the significant differences of each group’s data, and the reaction time (RT), the standard deviation of speed (SDS), and the difference of average velocity ($\Delta \bar{v}$) with large significant differences were determined as the cluster indicators. The descriptions of the three indexes are shown in Table 2. K-means clustering method was carried out to obtain the thresholds of the three indexes corresponding to different personality traits.

#### 4.1.1. Analysis of Driving Characteristics Based on K-Means Clustering Algorithm

**Figure 5:**
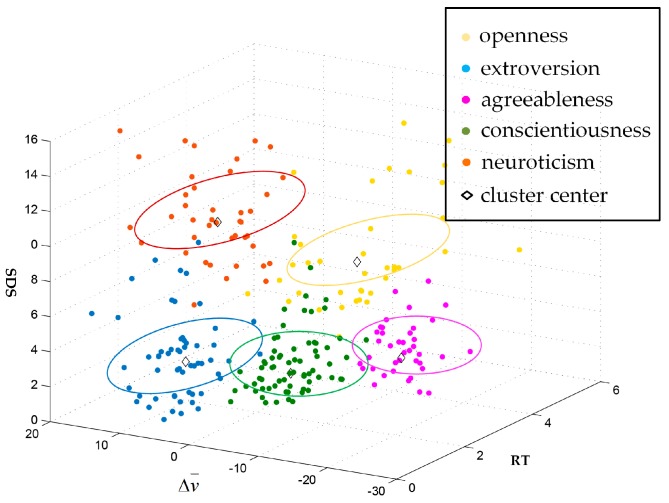
The results of the K-means clustering analysis. Note: RT: response time; SDS: standard deviation of speed; $\Delta \bar{v}$: difference of average velocity.

According to the Big Five personality traits, the number of clusters k of the K-means clustering algorithm was set to five, meaning that based on the driving data, we divided the drivers’ personality states into five types. The clustering results are shown in Figure 5, and the basic characteristics of the five types of personality state are shown in Table 3.

We divided the five kinds of personality state into two segments according to the changes of SDS.

The first segment included conscientiousness, extroversion, and agreeableness, and the level of SDS was low. For conscientiousness, the difference of average velocity was small, while the reaction time was middling, indicating that the driving style was stable and rigorous, and the drivers tended to drive safely. The extroverted personality state had a strong response ability, but $\Delta \bar{v}$ was larger and in a positive trend, showing that drivers with this state were expert in interacting with their surroundings. Thirdly, the speed fluctuation associated with agreeableness was higher than that for the first two types of personality. $\Delta \bar{v}$ showed a negative trend, and the reaction ability was weak, illustrating that the driving style was gentle and not impatient, and the driving performance was friendly.

The second segment included neuroticism and openness, with high levels of SDS. $\Delta \bar{v}$ associated with neuroticism was positive and had a large range, while the reaction ability was strong. These observations indicate that drivers in neurotic states were highly susceptible to the influence of external situations, and there was a tendency towards reckless driving. For openness, the response ability was weak and the reaction time was long. However, the change range for $\Delta \bar{v}$ was lower than that for neurotic personality and tended towards negative development, illustrating that this kind of personality state was not sensitive to the driving environment.

#### 4.1.2. Analysis of Dynamic Personality Based on Personality Baseline

**Figure 6:**
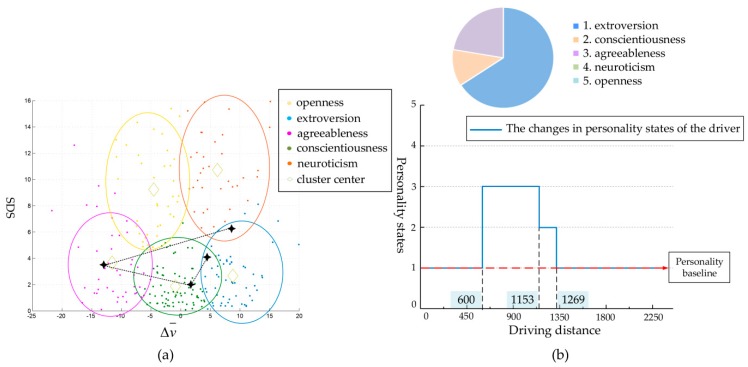
Changes in the personality states of the tested driver in the risk-free scenario. (**a**): The changing line of personality states in the driver’s continuous driving experiment; (**b**): Proportion diagram of five personality states in the risk-free scenario, and the changes of personality states with driving distance.

Based on the results of personality clustering in the previous section, we plotted the changes of the drivers’ personality states on a graph. Taking Driver 1 as an example, whose personality baseline was extroversion, we can see the changing line of personality states in the driver’s continuous driving experiment in Figure 6a. In the whole process, as shown in Figure 6b, the state of extroversion accounted for 66%, while conscientiousness and agreeableness accounted for 11% and 23%, respectively.

Specifically, the driver was in good spirits at the beginning of the driving experiment. As we can see from Figure 1, the first segment of the driving experiment was free driving, in which the internal speed limit was 60 km/h, and the dominant personality state was extroversion. The second segment was the speed limit stage, and the driver controlled the speed well in the state of agreeableness. When entering the lane-changing stage, the driver was supposed to interact with the surrounding vehicles. At this time, the driver became rigorous in driving and his personality state was conscientious. After the lane-changing was completed, the state returned to the baseline, i.e., extroversion, until the end of the driving experiment.

### 4.2. Risky Driving Scenarios

**Figure 7:**
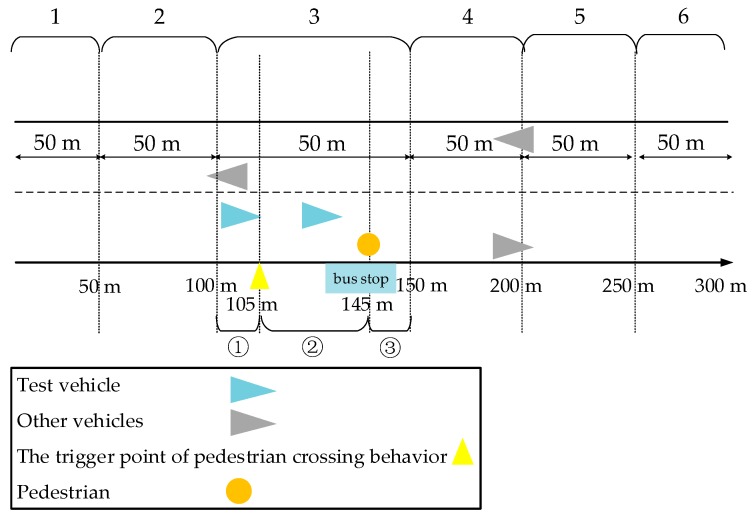
The segments of the risky scenarios.

The data recorded in the 150 m before and after the interaction between the experimental vehicle and the pedestrian were mainly analyzed. As shown in Figure 7, we divided the data into six 50 m segments, numbered 1 to 6. A description of each segment is shown in Table 4.

#### 4.2.1. The Traditional Approach to Personality in High- and Low-Risk Scenarios

After data processing, it was found that the indexes in Segments 2 to 5 showed significant changes. Therefore, we mainly analyzed the driving characteristics and changes of personality states in Segments 2, 3, 4, and 5 in this section.

**Figure 8:**
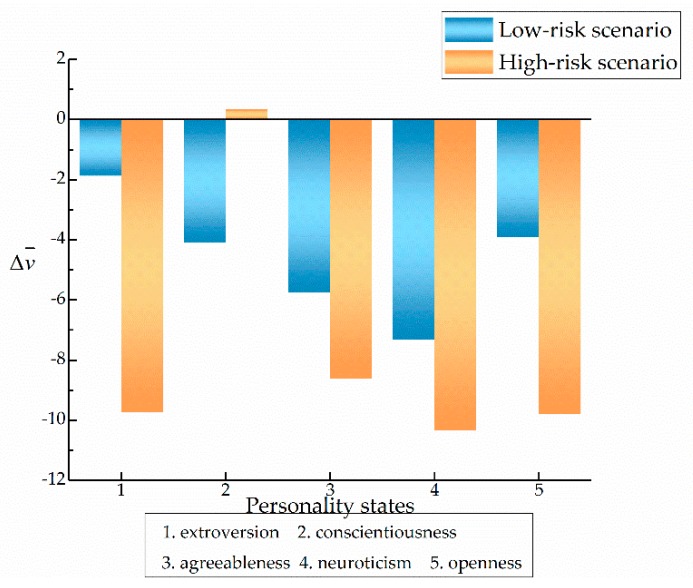
Comparison of $\Delta \bar{v}$ for the five types of personality in risky scenarios.

**Figure 9:**
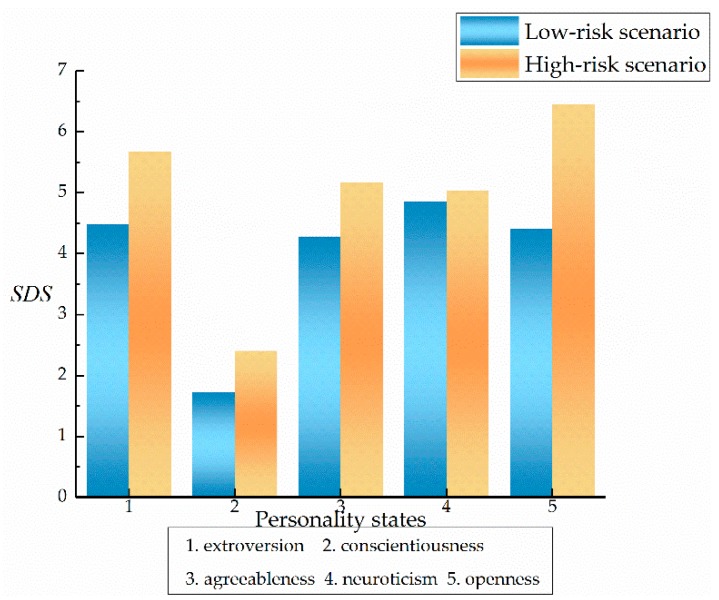
Comparison of SDS for the five types of personality in risky scenarios.

The traditional approach to personality was adopted to analyze SDS and $\Delta \bar{v}$ of the drivers in high- and low-risk scenarios, assuming that the personality states remained stable and unchangeable. The personality state of each driver was determined according to both the NEO-FFI scale and the driver’s performance in the risk-free driving scenario. Table 5 shows the calculated corresponding values of $\Delta \bar{v}$ and SDS for the five types of personalities in Segments 2, 3, 4, and 5. Due to the interaction with the pedestrian in Segment 3, where the driving characteristics of drivers changed to a large extent, we took segment 3 as an example for detailed explanation (Figure 8 and Figure 9).

Figure 8 shows the comparison of $\Delta \bar{v}$ in the high- and low-risk scenarios. The comparative analysis of the personality states showed that, except for the conscientious personality, the $\Delta \bar{v}$ of the other four types of personality was greater in the high-risk scenario. In addition, when calculating the difference of this index between high- and low-risk scenarios, for drivers with extroversion and openness, the index difference was 7.86 and 5.894, respectively, followed by conscientiousness, which was 4.468. However, there was little difference for agreeableness and neuroticism. In terms of inter-group (inter-personality), in the low-risk scenario, the average speed difference of drivers with extroversion was the lowest, while agreeableness and neuroticism scored higher than conscientiousness and openness. In the high-risk scenario, the $\Delta \bar{v}$ scores of the four types other than conscientiousness were higher.

Figure 9 shows the comparison of SDS in the two risk scenarios. In terms of intra-group comparison, the differential score of SDS for drivers with openness was the highest among the five types of personality (2.039). Extroversion followed with 1.186, while there was little difference in the performance of conscientiousness, agreeableness, and neuroticism states. From the comparison between groups, in the low-risk scenario, the speed of the conscientiousness state was much lower than that of the other four types of personalities, for which the performances were relatively consistent. In the high-risk scenario, the level of speed fluctuation associated with conscientiousness was slightly improved, but still lower than other personality states, while the open and extroverted personalities produced higher speed fluctuations. In summary, it can be seen that the high-risk situation provided a greater degree of stimulation for the drivers of all five types of personality, especially influencing the speed of the open and extroverted personalities.

By analyzing the values of each indicator, we found that in addition to the above conclusions, the values of the indexes in this section were not completely consistent with the threshold results obtained in the risk-free scenario. The reason, is that we assumed the personality states remained stable and unchangeable, and neglected that the behavior of pedestrian crossing the street may have had a certain impact on the personality states of the driver. This further illustrates that when studying driving behavior, we should examine the specific driving situation and study the driver’s personality and driving style from a dynamic point of view, so as to better understand the reasons for different driving reactions.

#### 4.2.2. Analysis of Dynamic Changes of Personality States in High- and Low-Risk Scenarios

In the analysis of the risk-free scenario, we found that during extended periods of driving, the driver may show a series of dynamic changes in personality states related to driving time and driving situation, such as intersections. Similarly, in the interaction with the pedestrian, even if the driving distance was short, the personality states fluctuated due to the different influences of high- and low-risk scenarios on drivers of different personality states. It can be seen from the above that Driver 1 had a more extroverted personality state in the risk-free scenario. Therefore, this section takes this driver as an example with which to analyze the changes of personality states of a driver with an extroverted personality baseline.

**Figure 10:**
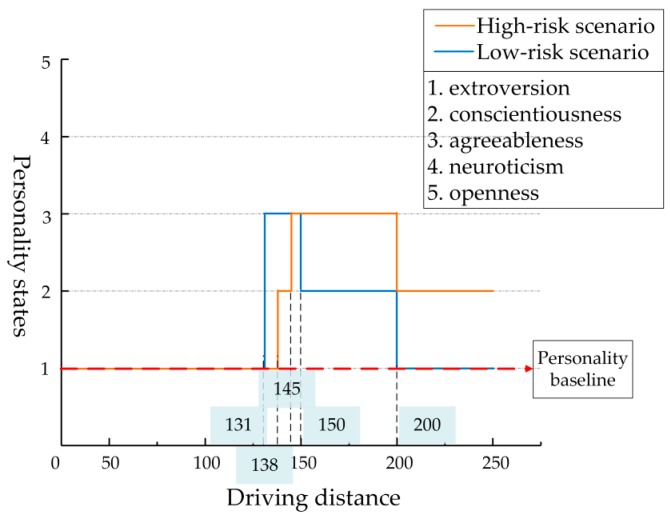
Changes of personality state in high- and low-risk scenarios based on driving distance.

**Figure 11:**
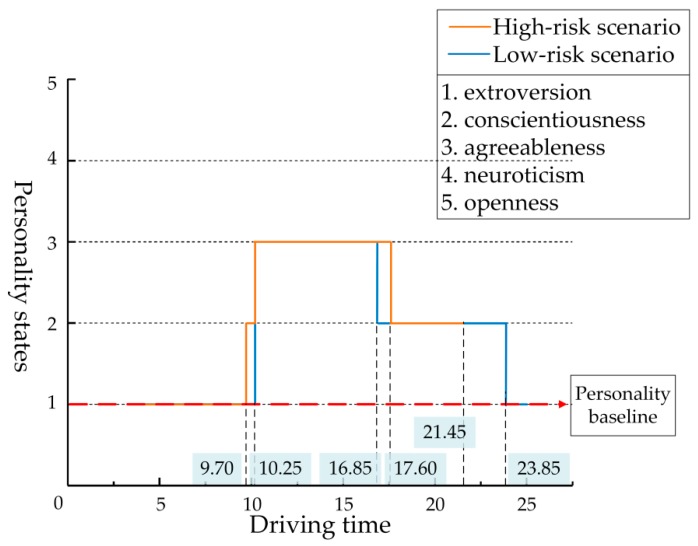
Changes of personality states in high- and low-risk scenarios based on driving time.

Figure 10 and Figure 11 show the personality state changes of Driver 1 in high- and low-risk scenarios related to the driving distance and driving time, respectively. Table 6 shows changes in the specific personality states and data of relevant indexes. This section includes data from both high- and low-risk scenarios.

In the high-risk scenario, when the driver reached 105 m (7.37 s), the pedestrian began to cross the street. As the bus obscured the driver’s optical line of sight, the tested driver could not see the pedestrian and continued to drive at a high speed. After 1.35 s, the pedestrian walked past the bus and came into the driver’s view. At this time, the driver reached 125 m and made the braking response quickly and cautiously at 138 m (9.70 s), where the personality state showed conscientiousness. Because of the high speed, the interaction distance and time between the pedestrian and the driver were shorter and the collision risk was higher than in the low-risk scenario. After adjusting the speed, the driver interacted with the pedestrian at 145 m; although the risk was high, there was no collision, and the personality state was agreeableness. Due to the intense driving, the driver maintained agreeableness to 200 m (17.60 s), then gradually returned to his normal state, and carefully accelerated to 250 m where the personality state was conscientiousness. The driver finally completed the driving at 21.45 s.

In the low-risk scenario, the pedestrian began to cross the street at 7.72 s, and the driver had a better line of sight because there was no obstruction in the scene. When the driver saw the pedestrian at 131 m (10.25 s), he was farther away from the pedestrian and traveling at a lower speed, and there was plenty of time to make the deceleration adjustment. In this process, the driver maintained his personality baseline, i.e., extroversion. Before the driver reached 145 m (15.35 s), the pedestrian had safely passed through the driving lane, so the interactive risk was low, and the driver’s personality state was agreeableness. The driver continued to drive, cautious in the process of acceleration due to the influence of pedestrian crossing behavior, and showed the state of conscientiousness. After entering Segment 5, the speed fluctuation value of the driver was low, the personality state returns to extroversion, and the driver reached 250 m at 28.57 s.

To sum up, it is not difficult to see that in the high-risk scenario, the blocking of view by the bus in front of the bus stop had a great impact on the driving behavior of drivers, and the changes of personality states were also more abundant. Therefore, we believe that in the same driving scene, when evaluating the reasons for different performances of drivers, the impact of specific situations on the drivers’ personality states in addition to driving ability and experience cannot be underestimated.

## 5. Conclusions

In this paper, we addressed the relationship between personality states and driving behavior from the dynamic perspective, and selected risk scenarios triggered by a pedestrian crossing the street as the research background. We identified three indicators to characterize personality, and K-means clustering algorithm was used to obtain thresholds for each index. On this basis, we analyzed the dynamic changes of personality states in the risk scenes.

The results showed that even in the risk-free scenario, specific driving operations such as car-following and lane-changing can still exert influence on personality state, which is in line with the theory of dynamic personality. When the changes of personality states in risky scenarios were compared, the results showed that in the high-risk scenario, the driver’s personality states changed more abundantly, and the time required to restore the personality baseline was also longer.

This paper provides a novel idea for the analysis of driving behavior, which fully combines the dynamic change of personality states with analysis of the driving environment. After analyzing the changing patterns of personality states, fundamental education could be provided for drivers to reduce the proportion of risky driving behavior and improve road safety.

Although this paper involved extended research, there were still some limitations that should be acknowledged. We considered two risk scenarios, but there are many other risk scenarios that were not considered. We will analyze the impact of risk degree of scenarios on personality states in the future. In addition, because of the small sample size, the representative change rules of the five kinds of personality state could not be summarized. Due to the short test, a potential bias could have been introduced by individual learning processes. In future research, it will be necessary to expand the sample coverage and to extend the experimental period.

## Figures and Tables

**Table 1:** Big five personality and corresponding characteristics.

Personality	Characteristics
Neuroticism	Anxiety, hostility, and impulsiveness [44]
Extroversion	Excitement seeking, activity, and warmth [44]
Openness	Fantasy, actions, and ideas [44]
Conscientiousness	Order, dutifulness, and self-discipline [45]
Agreeableness	Trust, altruism, and compliance [45]

**Table 2:** Descriptions of the cluster indexes.

Index	Symbolic Representation	Description
Response time	RT	This index was used to represent the driver’s reaction ability. The reaction time specifically refers to the time interval experienced by the driver from the time of the start of the stimulation to the time when he makes response. For example, the reaction time can refer to the time interval from the time when the brake light of front vehicle is on to when the driver steps on the brake pedal.
Standard deviation of speed	SDS	This indicator was used to characterize the degree of speed fluctuations in each segment, specifically the standard deviation.
Difference of average velocity	$\Delta \bar{v}$	This indicator was used to characterize the difference in mean velocity between each segment and the entire driving process.

Note: RT: response time; SDS: standard deviation of speed; $\Delta \bar{v}$ difference of average velocity.

**Table 3:** The basic characteristics of five types of personality states.

K-Mean	Center of the Cluster			Minimum/Maximum of the Indexes		
Personality	RT	SDS	$\Delta \bar{v}$	RT	SDS	$\Delta \bar{v}$
Conscientiousness	2.570	2.342	−1.947	2.15/3.91	0/6.787	−4.941/4.749
Extroversion	2.043	3.862	8.962	0.6/2.81	0.625/8.956	4.876/16.189
Agreeableness	2.987	2.797	−9.107	3.45/5.1	0.842/14.031	−15.457/−5.007
Neuroticism	2.525	12.543	11.392	0.8/3.03	7.672/15.497	−4.032/18.607
Openness	3.314	10.945	−1.529	2.8/5.44	6.579/12.864	−9.762/4.897

**Table 4:** Description of each segment in risk scenarios.

Segment	Description
1	The tested driver starts to drive with speed limit of 50 km/h
2	Maintain or adjust speed
3	① When the driver reaches 105 m, the pedestrian starts to cross the street ② The interaction stage between driver and pedestrian. The driver adjusts driving behavior to Avoid collision ③ The interaction is completed, and the driver continuously slows down or maintains the original behavior
4	Return to acceleration
5	Continue driving
6	Continue driving

**Table 5:** Values of driving indexes in each segment for high- and low-risk driving scenarios.

Index	Segment	Extroversion		Conscientiousness		Agreeableness		Neuroticism		Openness	
		Risk Scenario		Risk Scenario		Risk Scenario		Risk Scenario		Risk Scenario	
		Low	High	Low	High	Low	High	Low	High	Low	High
Difference of average velocity ($\Delta \bar{v}$)	2	9.89	11.774	5.111	9.565	15.742	14.117	12.475	12.913	12.45	15.742
	3	−1.874	−9.734	−4.083	0.358	−5.759	−8.607	−7.324	−10.332	−3.9	−9.794
	4	−12.597	2.221	−0.542	−11.67	−4.43	2.65	−2.009	0.843	−8.328	−2.192
	5	7.695	15.208	6.123	1.032	5.964	13.488	11.981	17.24	7.579	15.832
Standard deviation of speed (SDS)	2	0.741	0.657	2.082	0.589	0.594	1.356	0.301	4.669	0.76	0.829
	3	4.483	5.669	1.722	2.403	4.28	5.166	4.86	5.035	4.413	6.452
	4	9.254	8.073	2.542	6.566	6.085	8.135	8.638	8.501	8.576	7.12
	5	1.77	1.062	2.331	2.982	1.534	1.941	1.574	1.867	1.996	2.529

**Table 6:** Changes in personality states and data of relevant indexes in high- and low-risk scenarios.

Scenario	Segment		Distance	Time	Personality State	SDS	Average Speed	Max. Speed	Min. Speed
High-risk scenario	2		100	3.5–7.05	Extroversion	0.565	51.475	52.402	50.571
	3	②	**138**	**9.70**	Extroversion	0.366	52.227	52.593	51.231
			**145**	**10.25**	Conscientiousness	3.819	45.100	50.410	39.037
		③	**150**	**10.77**	Agreeableness	4.522	30.544	37.247	23.750
	4		**200**	**17.60**	Agreeableness	9.306	26.815	43.303	13.210
	5		250	**21.45**	Conscientiousness	1.960	47.052	50.385	43.509
Low-risk scenario	2		100	3.57–7.25	Extroversion	1.858	48.358	49.426	42.766
	3	②	**131**	**10.25**	Extroversion	1.598	38.307	42.498	36.860
			**145**	**15.35**	Agreeableness	9.217	9.178	36.161	0.000
		③	**150**	**16.85**	Agreeableness	1.137	12.355	14.248	10.451
	4		**200**	**23.85**	Conscientiousness	6.679	25.719	34.637	14.389
	5		250	**28.57**	Extroversion	1.826	38.339	40.839	34.720

Note: Bold indicates the time taken for the driver to complete each key driving segment when driving in high- and low-risk scenarios. Segments “2”to “5” correspond to the specific segments in the risk scenario respectively, and both sub-segments ② and ③ belong to the segment 2. The description of each segment can be seen in Table 4.
